# Smaller = Denser, and the Brain Knows It: Natural Statistics of Object Density Shape Weight Expectations

**DOI:** 10.1371/journal.pone.0119794

**Published:** 2015-03-13

**Authors:** Megan A. K. Peters, Jonathan Balzer, Ladan Shams

**Affiliations:** 1 Department of Psychology, University of California Los Angeles, 1285 Franz Hall, Box 951563, Los Angeles, California, 90095–1563, United States of America; 2 Department of Computer Science, University of California Los Angeles, 4732 Boelter Hall, Los Angeles, California, 90095, United States of America; 3 Department of Bioengineering, University of California Los Angeles, 420 Westwood Plaza, 5121 Engineering V, Los Angeles, California, 90095–1600, United States of America; Universitat de Valencia, SPAIN

## Abstract

If one nondescript object’s volume is twice that of another, is it necessarily twice as heavy? As larger objects are typically heavier than smaller ones, one might assume humans use such heuristics in preparing to lift novel objects if other informative cues (e.g., material, previous lifts) are unavailable. However, it is also known that humans are sensitive to statistical properties of our environments, and that such sensitivity can bias perception. Here we asked whether statistical regularities in properties of liftable, everyday objects would bias human observers’ predictions about objects’ weight relationships. We developed state-of-the-art computer vision techniques to precisely measure the volume of everyday objects, and also measured their weight. We discovered that for liftable man-made objects, “twice as large” doesn’t mean “twice as heavy”: Smaller objects are typically denser, following a power function of volume. Interestingly, this “smaller is denser” relationship does not hold for natural or unliftable objects, suggesting some ideal density range for objects designed to be lifted. We then asked human observers to predict weight relationships between novel objects without lifting them; crucially, these weight predictions *quantitatively* match typical weight relationships shown by similarly-sized objects in everyday environments. These results indicate that the human brain represents the statistics of everyday objects and that this representation can be quantitatively abstracted and applied to novel objects. Finally, that the brain possesses and can use precise knowledge of the nonlinear association between size and weight carries important implications for implementation of forward models of motor control in artificial systems.

## Introduction

Even advanced artificial systems cannot grasp and lift objects with ‘human-like’ ease and dexterity. Theories of sensorimotor processing recognize that our ability rests not just on fast or precise sensory responses to errors, but also on our accurate and precise *predictions of* the sensory consequences of motor commands [1–3]. Yet what is the basis for predictions about an object’s weight? Often, we use visual information about size, shape, and material (density), as well as memory of previous lifts [4–9]. Yet if an object’s material is uninformative and it has never been lifted before, is a visual estimate of size and shape enough to predict weight correctly? While it is known that human observers expect larger objects to be heavier than smaller ones [5,8–10], the quantitative precision of this estimation is unclear. Do we use a simple heuristic—e.g., that an object with twice the volume should be twice as heavy—or is a more complex calculation involved? We aimed to explore whether knowledge of environmental statistics may play a role in weight prediction.

A wealth of data demonstrates that humans are sensitive to statistical environmental properties: Light generally comes from above, leading to strong perceptions of convexity in shaded 2D objects [11]; motion in the world is typically slow and smooth, biasing humans’ visual estimates of speed under uncertain conditions [12,13]; environmental distributions of contour orientation cluster around cardinal directions, biasing perception [14]; and human observers are also biased to perceive that objects are convex and background colors are homogenous [15] due to regular patterns in these environmental properties. Thus, it is clear that the human brain can maintain representations of environmental regularities.

How are such representations obtained? Many studies have demonstrated that pure exposure to statistical regularities (e.g., regular pairing of sensory stimuli) in an experimental environment can lead to learning [16–18]. Recent studies have also begun to explore how humans can learn simultaneous, independent statistics of multimodal inputs [19] as well as cross-modal associations between audio-visual cues [20]. However, although participants can learn joint statistical properties of two simultaneous distributions within the visual modality [21], it remains unclear how the brain may represent a distribution of the *co-occurrence* of *crossmodal* environmental properties [22]. And although it has been suggested that humans can learn qualitative statistics of object weights and sizes in an artificial setting [6], the degree to which the brain can extract an abstract representation of the typical link between object size and weight through heterogeneous everyday experience is not known. It is also unknown whether any such representations might contain quantitative features. We hypothesized that, given the statistical learning abilities of the brain shown in other contexts, there may be a similar learning phenomenon operating here that would extract the relationship between volume and weight for the objects humans regularly lift and manipulate and make it available to the perceptual system, even before an object is lifted.

## Materials and Methods

### Environmental data collection

To identify the true relationship between volume and weight in everyday environments, three datasets of artificial, liftable objects were collected. For Dataset 1 in S1 Dataset, using a ruler and basic geometry, we estimated the volume of a set of 43 objects selected randomly from everyday home and office environments. Examples of objects used include computer mice, smartphones, shoes, coffee mugs, staplers, cooking utensils, packaged food items, and personal care items such as soap and shampoo. In the interest of efficiency, we next turned to a coarser measure to supplement the objects we had sampled from homes and the office environment, seeking out basic product dimensions (length, width, height, and weight) available on online shopping sites such as Amazon.com and other online retailers. Such coarse information was collected for 124 household objects and made up Dataset 2 in S2 Dataset. Although this method of measurement is coarse, it allowed us to sample a much broader set of objects than would have been possible had we been restricted to our own homes and offices, and without necessitating purchasing, shipping or transporting, and storing the items.

Finally, to gain a more precise estimate of volume than what is provided by tape measurements or online surveys, we developed a custom software package. Video and point-depth estimates captured by a Carmine Primesense 1.09 depth sensor were fed into a depth-estimation algorithm and used to produce a mesh grid virtual representation of 28 man-made household objects [23]. These objects’ volumes were calculated from the mesh grid virtual representations through our custom software written in Qt Creator and Matlab, and used to generate Dataset 3 in S3 Dataset [23]. We applied the same method to generate Dataset 4 in S3 Dataset, which consisted of 28 natural objects, such as fruits, vegetables, and objects found in the outdoors (e.g., pinecones).

The method developed enables precise measurements of the volume of everyday objects in a user-friendly, inexpensive manner. Note that such objects often exhibit complex geometry, topology, and photometry, thus precluding the use of off-the-shelf laser scanners (due to specular reflections); volume displacement techniques, e.g., submerging objects in water, cannot be easily employed as many objects either float (e.g., apples), absorb water (e.g., cardboard packaging for foodstuffs, stuffed animals), or are permanently damaged by water (e.g., hand-held consumer electronics). Further, we wished to measure volume in a manner as analogous as possible to the way in which humans do so without access to haptic information, i.e., on the basis of visual information alone [24]. For example, visual inspection prior to lifting would provide no information about internal cavities (as in hollow or porous objects). Thus, we applied these state-of-the-art Computer Vision algorithms to produce 3-D models of everyday man-made (Dataset 3 in S3 Dataset) and naturally-occurring (Dataset 4 in S3 Dataset) objects [23] and calculated their volumes and densities. Our method has been tested and validated, and produces an average relative volume error of-0.34%, making it both accurate and precise [23]. The software is freely available for download at https://bitbucket.org/jbalzer/yas/wiki/Home.

For all objects in Datasets 1 in S1 Dataset, Datasets 3 and 4 in S3 Dataset, objects’ weight was measured to 0.1g precision using an electronic scale (American Weigh). A final dataset was constructed via online survey as for Dataset 2 in S2 Dataset (i.e., gathering length, width, height, and weight as reported on product pages) for 28 artificial but *unliftable* objects, such as large furniture, large household appliances, and vehicles (Dataset 5 in S2 Dataset).

### Perceptual Experiment

**Human subjects.** Twenty individuals (mean age: 19.9 years, range: 18–25 years, 3 men, 16 right-handed) gave written informed consent to participate in the perceptual portion of this study. All participants had normal or corrected-to-normal vision and normal hearing.

**Ethics statement.** This experiment was conducted in accordance with the Declaration of Helsinki and approved by the UCLA Institutional Review Board.

**Materials.** Experimental stimuli consisted of twelve objects: three sets of four objects possessing identical volume ratios. From small to large, these objects will be referred to as objects A, B (3.375 times A’s volume), C (8 times A’s volume), and D (27 times A’s volume). The Blob set consisted of four identically-shaped blobs spray-painted blue, with volumes of 111.63, 376.75, 893.03, and 3013.98 cm^3^, respectively. The Greeble set consisted of four identically-shaped greebles spray-painted green, with volumes of 65.72, 221.80, 525.75, and 1774.41 cm^3^, respectively. The Blob and Greeble sets were constructed via 3-D printing out of a plaster-like substance. The Cube set consisted of four cubic objects constructed out of tagboard and covered in balsa wood, with volumes of 131.10, 442.45, 1048.77, and 3539.61 cm^3^, respectively. All objects were hollow. Objects not in use on a given trial remained hidden behind a black curtain; the experimenter also remained hidden from view.

**Perceptual task procedure.** Subjects were randomly assigned to one of two groups: The Expected Weight (EW) group was given instructions to report their expectation about weight, while the Perceived Volume (PV) group was given instructions to report their perception of volume. Groups were comparable in terms of demographic composition. On each trial, objects were presented two at a time, placed side by side in front of the participant on a black cloth (so as to dampen any sounds associated with their placement that might be used as cues to density). The object to the participant’s left was given a reference value [25,26] of 10 units (units of weight for the EW group, and units of volume for the PV group), and the subject was instructed to verbally report his expectation regarding the object on the right, in the form of a ratio referencing the left object’s value of 10 units. For example, if a small object was presented on the left, and a larger one on the right, a subject in the EW group might say “20” if he believed the larger object should weigh twice as much as the smaller; a subject in the PV group might say “30” if he believed the larger object possessed three times the volume of the smaller; and so on. Likewise, if the right object was smaller than the left, a subject might say “5” to indicate his belief that the right object possessed half the volume or weight as the left one. Subjects were instructed to provide this report without touching, lifting, or moving the objects in any way.

Objects were presented in a full factorial design, including all six possible combinations of the four sizes for each object. Thus, the possible pairings within each object were: A:B, A:C, A:D, B:C, B:D, C:D, (small/left—large/right, S-L); and B:A, C:A, D:A, C:B, D:B, D:C (large/left—small/right, L-S). Subjects completed 10 practice trials, followed by 144 test trials (10 trials of each S-L pairing, 10 trials of each L-S pairing) in pseudorandomized order. No feedback was given. While the experimenter was placing or removing the objects, subjects in both groups were required to close their eyes so as to avoid any cueing effects regarding the possible weight of the objects. The experimenter monitored compliance with all instructions through a small slit in the black curtain.

For analysis, we collapsed across S-L and L-S orderings within an object type; for example, data from the A:C and C:A conditions were pooled for each subject to create a single dataset representing this pair of objects, regardless of presentation placement.

### Statistical analyses

All analyses for both environmental and perceptual data were carried out through the use of the Matlab software (Version 7.10.0) with the Statistics Toolbox and the SPSS Statistics software (Version 20.0.0). Means and standard deviations were calculated after taking the natural log transform of each data point to restore linearity in responses, as responses were made as ratios, which are distributed nonlinearly. For some plots and tables, data are transformed back into ratio form for ease of interpretation. Sample size of n = 10 in each group was determined to be sufficient given the identification of a medium effect size (Cohen’s *d*) in a pilot experiment; after reaching n = 10 in each group, data collection was terminated. All data are available for download as Supplemental Material.

## Results

### Environmental object data

**Fig 1:**
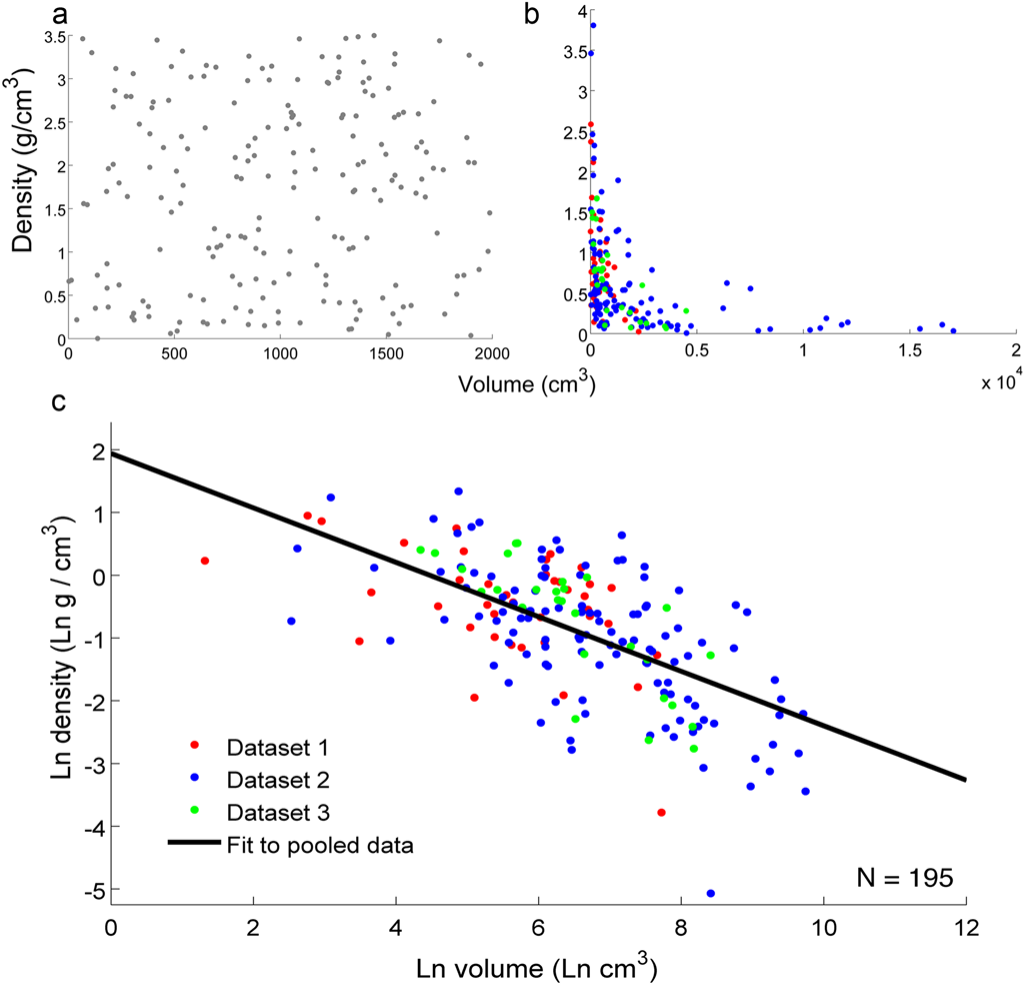
Environmental data. ‘Uniform distribution’ predictions (a) differ markedly from the observed power function relationship between volume and density (b). For ease of viewing, (c) displays the natural log-transformed scatterplot of the power function relationship between volume and density for man-made objects, showing a significant inverse correlation between log volume and log density.

**Fig 2:**
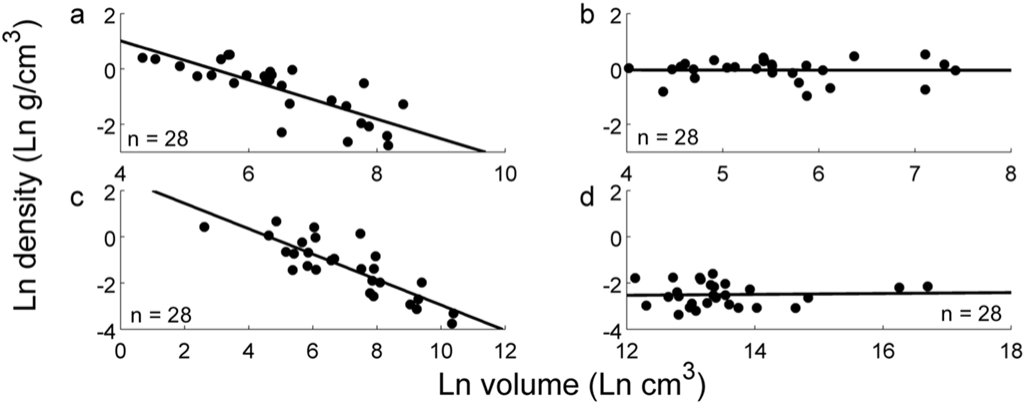
Comparison of density distribution for natural, artificial, liftable, and unliftable objects. (a) 3-D scanned liftable artificial objects (Dataset 3 in S3 Dataset, n = 28) show a significant inverse correlation between log volume and density, while (b) 3-D scanned natural objects (Dataset 4 in S3 Dataset, n = 28) show no such relationship. Likewise, (c) a subset of randomly-selected objects from the liftable artificial objects collected via online survey (random subset of Dataset 2 in S2 Dataset, n = 28) also demonstrate this significant inverse correlation, but (d) artificial but *unliftable* objects collected via online survey (Dataset 5 in S2 Dataset, n = 28) show no correlation.

True volume and weight data was collected for 195 liftable, man-made, everyday objects, and used to calculate their density: *d = w/V* where *d* is density, *w* is weight, and *V* is volume. Technically, *d = m/V*, where *m* is mass; however, because *w = m × a*, where *a* is acceleration (in this case, acceleration is due to gravity, which is constant), and because weight and mass are used interchangeably in everyday discourse, weight is used as a functional equivalent to mass in this experiment. We used the property of density because it is defined as the very relationship we were interested in (that between volume and weight) and density estimation is often mentioned as a crucial factor in preparation for lifting objects [5]. In contrast to predictions of independence between volume and density (Fig. 1a), a power function relationship between volume and density was observed for the man-made object datasets (Dataset 1 in S1 Dataset and Dataset 2 in S2 Dataset and Dataset 3 in S3 Dataset) (Fig. 1b), so a log transform was computed to reveal the nature of the inverse correlation between volume and density for each of the three man-made object datasets (R_1_ = -.4673, p = .002; R_2_ = -.6290, p << .001; R_3_ = -.7917, p << .001), as well as the pooled man-made object data (R = -.5721, p << .001) (Fig. 1c). To compare directly between artificial and natural objects, the same calculation was also performed for a dataset of natural, liftable objects (Dataset 4 in S3 Dataset) (R_4_ = -.0048, p = .981), but revealed no significant relationship between volume and density (Fig. 2a and 2b). A final comparison between a randomly-selected subset (n = 28) of objects in Dataset 2 in S2 Dataset (liftable artificial object statistics garnered from online retailers) and a set of *unliftable* artificial objects with dimensions and weight data collected in the same manner (Dataset 5 in S2 Dataset) also revealed the persistence of the inverse correlation for the subset of liftable artificial objects (R_2,subset_ = -.8390, p << .001) but not the unliftable ones (R_5_ = .0396, p = .8416) (Fig. 2c and 2d). Thus, these data revealed that, for liftable man-made objects, density is distributed not uniformly, but instead as a power function of volume: Smaller liftable artificial objects are denser than larger ones, and by more so the smaller they are. This relationship does not hold for natural objects or unliftable man-made objects. (See also S1 Fig. for non-log-transformed data.)

### Perceptual Experiment

Participants were shown pairs of similarly-shaped but differently-sized objects, and asked to judge their weight ratio (Expected Weight group; EW) or volume ratio (Perceived Volume; PV group) (See Materials and Methods). If the brain had no knowledge or representation of the environmental statistic linking objects’ weights and densities to their size, answers from participants in the two groups should be identical, on average: Without density information, two objects’ weight ratio should simply be their volume ratio. A difference between group answers would indicate that observers are relying on additional information about objects’ densities to form their weight expectation judgments.

Due to the nature of the dependent measure as a ratio for the Expected Weight (EW) and Perceived Volume (PV) groups (and in keeping with studies on relative mass in intuitive physics [27]), the natural log transform of each data-point was computed, as was the mean log ratio for each subject for each cube pair. Normality of each of these resultant datasets was then assessed through the Lilliefors test [28]—an adaptation of the Kolmogorov-Smirnov one-sample test that allows for testing the null hypothesis that data come from a normally distributed population without the need to specify the expected value and variance of the null hypothesis test distribution. No distributions failed these normality tests.

**Fig 3:**
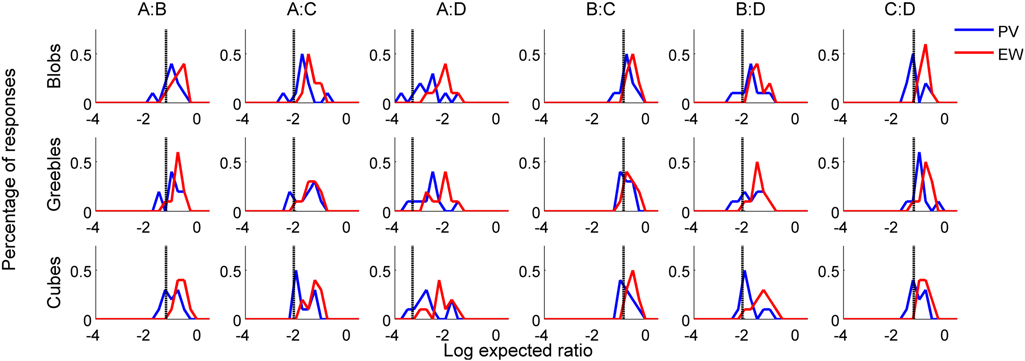
Human observers’ data, by condition and object type. Participants’ reported PV ratios are consistently smaller than EW responses, indicating a belief that smaller objects are denser than larger ones. Consistent with previous studies, PV consistently underestimates true volume, leading to PV responses larger than the true volume ratio between the objects (gray vertical line). EW ratios are consistently larger than PV ratios, indicating that subjects believe smaller objects are denser than larger objects, over and above any mis-estimation of volume.

**Fig 4:**
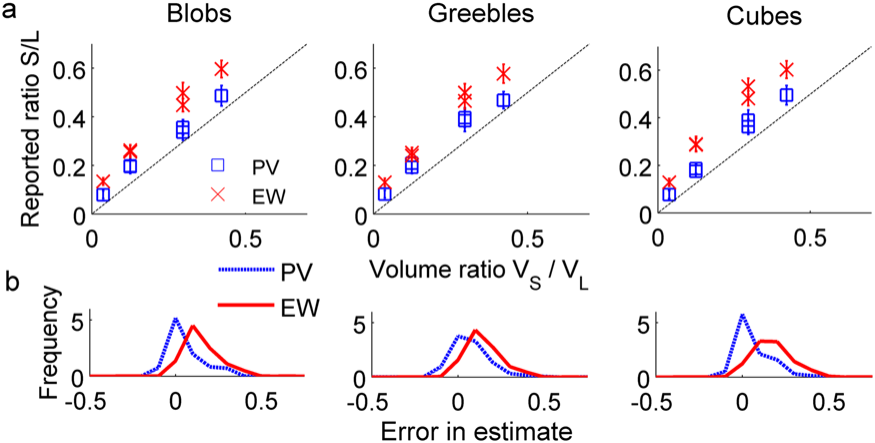
Human observers’ data, summary. (a) As before, EW and PV responses for each object type by pair show the “smaller is denser” belief, with EW responses consistently larger than PV responses. (b) Error in estimates (PV—true volume and EW—true volume) collapsed across all pairs demonstrates the effect of condition: EW ratios are larger than PW ratios, and thus display more error in comparison to true volume ratios. Error bars represent standard deviation of responses. The x-axis represents error in estimation of volume/weight.

Consistent with previous studies [29], PV ratios did not approach true volume ratios, indicating consistent underestimation of volume (two-tailed t-tests against 0: t_Blobs_ = 4.766, p << .001; t_Greebles_ = 5.4994, p << .001; t_Cubes_ = 5.1265, p << .001). We next conducted a 2 (condition: EW vs. PV) x 3 (object type: Blobs, Greebles, Cubes) x 6 (pair: A:B, A:C, A:D, B:C, B:D, C:D) mixed design ANOVA. This analysis revealed a main effect of condition (F(1,18) = 7.542, p = .013) and pair (F(5,90) = 334.179, p < .001), and an interaction between condition and pair (F(5,90) = 3.334, p = .008), but no other significant effects (p > 0.05). The main effect of condition indicates that participants in the EW group consistently reported larger ratios than did participants in the PV group; the direction of this effect indicates that observers believed the smaller objects to be denser than the larger objects—*over and above the typical underestimations of volume*—which qualitatively matches the statistics of the environment. The main effect of pair indicates that participants reported different EW and PV ratios for the pairs of objects, and the interaction indicates that the degree to which EW ratios were larger than PV ratios varies by pair (Figs. 3 and 4).

To further explore the interaction between pair and condition, we conducted six additional post-hoc 2 (condition: EW vs. PV) x 3 (object type: Blobs, Greebles, Cubes) mixed design ANOVAs, one for each object pair, to assess the degree to which the belief that smaller items are denser than larger ones persists for all pairs. Correction for multiple comparisons was accomplished through the False Discovery Rate method [30,31], which indicated that the expected percent of false predictions would be less than 0.2% for each of these six tests (Table 1). This result indicates the belief that smaller objects are denser than larger objects exists for all pairs individually, and that it is not one or two individual pairs that drive the overall main effect. No other significant effects were detected with these post-hoc tests. We also measured effect size (Cohen’s *d*) for each pair, collapsing across object set. This analysis revealed that effect size grew roughly with increasing dissimilarity between the two object volumes: *d*_*A*:*B*_ = 1.0480, *d*_*A*:*C*_ = 0.9780, *d*_*A*:*D*_ = 1.2134, *d*_*B*:*C*_ = 0.9816, *d*_*B*:*D*_ = 1.0037, *d*_*C*:*D*_ = 1.1835. All of these effect sizes are considered large. We also measured the effect size for each object set collapsing across pair, which revealed the Greebles object set effect size (*d*_*Greebles*_ = .4059) to be smaller than the other two object sets (*d*_*Blobs*_ = .5210, *d*_*Cubes*_ = .5819).

**Table 1:** Results of six post-hoc mixed design ANOVAs.

Pair	F_Condition_	p	FDR
**A:B**	5.945	0.025	0.0017
**A:C**	5.389	0.032	0.0013
**A:D**	8.441	0.009	0.0019
**B:C**	4.796	0.042	0.0015
**B:D**	5.441	0.031	0.0016
**C:D**	7.854	0.012	0.0013

Results demonstrate that the belief that smaller items are denser than larger ones exists for all pairs of objects in our experiment. Correction for multiple comparisons through use of the False Discovery Rate method indicates that false discovery is highly improbable, at less than 0.2% for each of the six tests.

### Comparison of environmental and perceptual data

Finally, we sought to assess the degree of *quantitative* agreement between the environmental and perceptual (EW) data in order to determine the nature of the representation our participants were using. Data were pooled from all liftable artificial object environmental datasets (Datasets 1–3 in S1–S3 Datasets), and a full factorial combination set of all volumes of all artificial objects was created. We then selected the half of the full factorial combination set for which *V*_*object1*_ *< V*_*object2*_, e.g. cases where *object 1*: *"9V battery"*, *object 2*: *"orange"* and not *object 1*: *"orange"*, *object 2*: *"9V battery"*.

**Fig 5:**
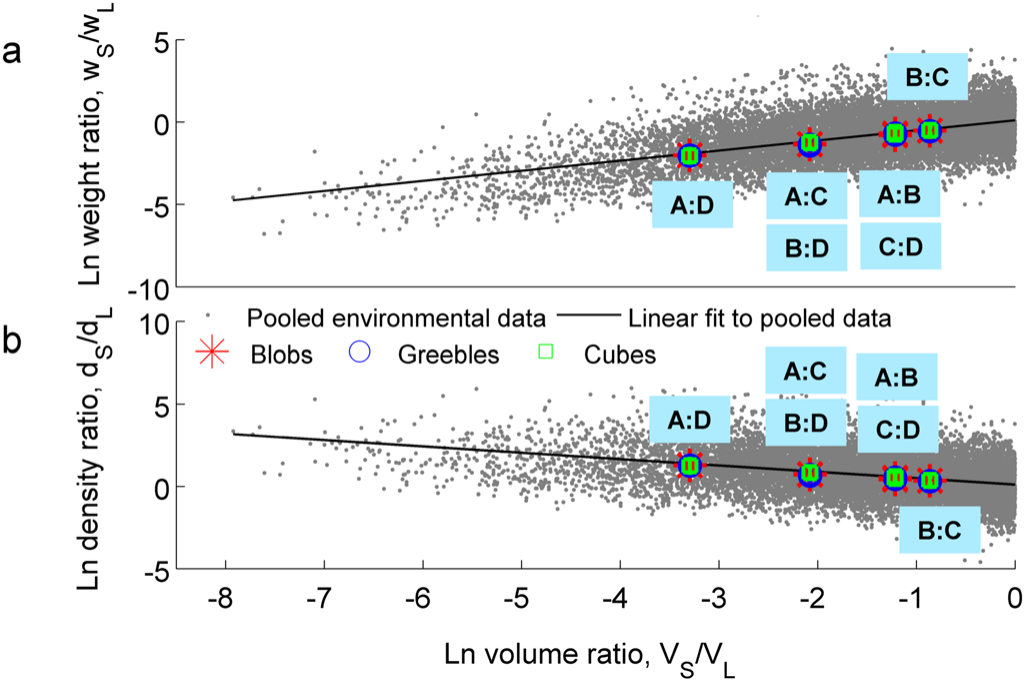
Comparison between environmental object data and observers’ data. Overlay of natural log-transformed environmental and observers’ expected (a) weight (EW) ratios and (b) density ratios as a function of volume ratios for the three object types shows agreement between environmental data and participants’ predictions of objects’ weight (and thus density) relationships. Error bars denote standard deviation across participants’ responses.

Next we computed the true *small/large* ratio for weight (*w*_*S*_ */w*_*L*_) and density (*d*_*S*_ */d*_*L*_) for each of these small-large object pairs, and, due to the identified power function relationships, computed their natural log transforms. Linear trends to the log environmental object weight ratio (WR) and density ratio (DR) data were fitted as a function of log volume ratios (VR) (WR = .613VR + .114, DR = -.387VR + .114) (Fig. 5). These linear trends were subsequently used to calculate the average log weight and density ratios for each of the volume ratios used in the perceptual experiment (Tables 2 and 3).

**Table 2:** Predicted weight ratios derived from line of best fit to environmental object data for the six volume ratios presented experimentally.

Pair	Predicted environmental *w*_*S*_ */w*_*L*_	EW ratios (Cubes)	EW ratios (Blobs)	EW ratios (Greebles)
**A:B**	0.5314	0.5325	0.4980	0.4654
**A:C**	0.3129	0.2867	0.2621	0.2520
**A:D**	0.1484	0.1282	0.1337	0.1302
**B:C**	0.6600	0.6027	0.5962	0.5769
**B:D**	0.3129	0.2895	0.2564	0.2402
**C:D**	0.5314	0.4816	0.4478	0.4992

**Table 3:** Predicted density ratios derived as in Table 2.

Pair	Predicted environmental *d*_*S*_ */d*_*L*_	*d*_*S*_ */d*_*L*_ (Cubes)	*d*_*S*_ */d*_*L*_ (Blobs)	*d*_*S*_ */d*_*L*_ (Greebles)
**A:B**	1.7933	1.7972	1.6809	1.5706
**A:C**	2.5036	2.2938	2.0967	2.0157
**A:D**	4.0068	3.4620	3.6092	3.5146
**B:C**	1.5643	1.4286	1.4133	1.3675
**B:D**	2.5036	2.3163	2.0514	1.9218
**C:D**	1.7933	1.6254	1.5113	1.6849

Finally, to compare these ratios with perceptual data, we calculated the expected density ratio for each EW data-point by again using the relationship *d = w/V*:

wSwL*1VS1VL=wSwL*VLVS=dSdL (1)

As the volume measurements for the environmental objects database are true volumes, true volume (as opposed to perceived volume) was used for these calculations as well. To compare to the environmental data, we computed the natural log transform of the resulting weight and density ratios. Results of this set of analyses are shown in Tables 2 and 3, transformed back into ratio space for ease of interpretation. Surprisingly, predicted weight ratios closely mirror true weight ratios in the environment (Fig. 5a), indicating that the amount by which observers expected a smaller man-made object to be denser than a larger one closely mirrored the average true density asymmetry for a similarly-sized pair of man-made objects in the environment (Fig. 5b). To confirm visual analysis, we computed the linear trends for the weight ratios (WR) and density ratios (DR) predicted from the perceptual experiment. This led to WR_Blobs_ = .622VR—.007, WR_Greebles_ = .638VR + .002, WR_Cubes_ = .646VR + .089, DR_Blobs_ = -.378VR—.007, DR_Greebles_ = -.362VR + .002, and DR_Cubes_ = -.354VR + .089, all of which closely match the calculated lines of best fit for the environmental object data. These findings suggest that the human nervous system is endowed with knowledge of and is able to use the power function relationship between size and density to optimally generate accurate estimates of novel, man-made objects’ weight relationships on the basis of visual size alone, even when other visual cues—such as differential material—and memory are unavailable.

## Discussion

In this study, we report a new environmental regularity: The distribution of liftable artificial object density follows a power function of volume, i.e., weight does not grow linearly with volume for objects that are designed to be liftable and manipulable. Furthermore, this statistical regularity does not appear to exist for natural objects that are liftable; a survey of larger artificial objects such as furniture and vehicles that are not designed to be liftable also did not show this relationship. These findings suggest that physiological constraints on humans’ lifting abilities (that the maximum comfortable liftable size and maximum comfortable liftable weight exist, but that as size increases maximum liftable weight decreases) have resulted in a set of everyday man-made objects that follows this unique power function between volume and density.

This environmental regularity appears to be encoded in the human sensorimotor system and used by the nervous system to predict novel objects’ weight relationships at a perceptually-available level. When shown pairs of novel objects and provided no informative cues to their weight relationship other than their visual appearance and no previous experience lifting any similar objects in the experimental setting, participants consistently and systematically provided weight estimates that indicated they believed smaller objects to be denser than larger objects, over and above any mis-estimations of volume. This effect was strong for the Blob and Cube object sets, but slightly less strong for the Greeble object set. It is likely this difference occurred because either: (a) the Greebles were smaller in volume; or (b) the Greebles possessed unique geometry (e.g., more cylindrical, protruding elements, etc.) in comparison to the other sets. It is also possible that the Greebles may have in part induced use of a prior of natural objects—which possess no regular size-density relationship—given that Greebles are designed to look somewhat animate. Indeed, several participants reported that the Greebles were “cute.” However, despite these possibilities, it still remains that three of the six Greeble pairs induced a significant density bias, and the effect in the remaining three was borderline significant.

Most strikingly, participants’ conscious estimates of a given two experimental objects’ weight relationships *quantitatively* match the average weight relationship held by two objects of similar volume relationship sampled from everyday environments: If a pair of objects in the environment displayed a density ratio of 2.5 on average, observers’ reports of expected weight ratio also drew upon an expected density ratio of about the same magnitude, rather than a simple qualitative relationship such as, “The smaller item should be somewhat denser than the larger.” These findings suggest that the human brain has learned quantitative aspects of the nonlinear relationship between size and weight for everyday objects, and can abstract that relationship in the absence of informative cues (e.g. to material) to a set of nondescript, novel objects that are to a certain extent consciously available. Although it has previously been demonstrated that the motor system possesses more quantitative information about a novel object’s weight, it has also been repeatedly found that the motor system and perceptual system are dissociable when it comes to lifting and manipulating objects: Even in the size-weight illusion, motor forces scale very quickly to correctly anticipate the weight of novel objects, and yet reports of weight expectations and weight perception do not [32–34]. Interestingly, when we informally yet explicitly asked participants whether they believed the two objects to possess the same density, many appeared confused by the question: Some said they were equally likely to possess equal or unequal density (i.e., 33% likely to have equal density, 33% likely the smaller was denser, 33% likely the larger was denser), while others reported rationalizations such as, “They appear to be made out of the same material, so they probably have the same density.” These comments indicate that although this quantitative “smaller is denser” information is to some extent consciously accessible, it nevertheless remains implicit to a certain degree.

These results first demonstrate that humans’ sensitivity to and use of environmental statistics can be extended to include joint distributions of properties, such as that linking size and weight. However, unlike many of the previously-reported environmental statistic sensitivities, our results additionally demonstrate that the sensorimotor system’s knowledge of the size-weight distribution (i.e., the distribution of density as a function of size) is represented *quantitatively* as well as qualitatively. Previous studies have demonstrated qualitative statistical sensitivities, or rules such as “slower and smoother motion is more likely” [12,13] or “more connected in space and time is more likely” [35,36]. In fact, humans’ qualitative acquisition of an experimentally-manipulated inversion of the relationship between size and weight has been demonstrated in a statistical learning study [6]. The authors presented observers with geometric stimuli uniform in material and color but of varying sizes, which had been constructed such that smaller objects were heavier than larger objects, in opposition to the typical direct relationship between size and weight. Results demonstrated that, with training, subjects’ produced motor forces came to demonstrate knowledge of this relationship: Eventually, subjects applied more grip and load force to smaller objects than to larger ones, suggesting their expectations that the smaller objects would be heavier.

It is important to note, however, that perceptual expectations of weight were not directly collected in this study, instead being inferred from reports of heaviness perception in the size-weight illusion. Because it is not yet settled how exactly heaviness perception depends on perceptual expectations of weight [5,32,37,38], it is difficult to draw specific conclusions about how exactly these perceptual expectations changed as a result of training with small-heavy and large-light objects. Further, this study suggested that the qualitative relationship between size and weight could be learned by experience, the learning was based on a set of objects that were uniform in shape (within a set), color, and material, and only varied in size and weight; it therefore remained unclear whether humans might be capable of such statistical learning in natural environments which pose extreme diversity of stimulus types (e.g., real-world objects). Further, and critically, the motoric force metric used in that study cannot speak to whether subjects learned only the qualitative inverted relationship between size and weight, or whether they learned a more quantitative representation: Recent evidence suggests that grip force, load force, and their first derivatives may reflect not only expectation of heaviness but also uncertainty (i.e., lack of confidence) about one’s expectation [39]. Additionally, while it has been shown that such forces scale directly with anticipated weight (including the integration of visual size cues in the anticipation of object weight) [4,6,9,40–42], the precise quantitative relationship between applied force and weight expectation (i.e., how many Newtons or Newtons/second reflect an expectation of how many grams) remains unclear. Verbal report thus serves as a purer measure of quantitative, perceptual expectations of weight relationships, and so was selected as the response measure for this study.

Thus, in contrast to these previous reports of qualitative learning, the current findings show that rather than simply relying on a heuristic-like rule that “smaller objects are typically denser” in the environment, or “objects in this setting have been manipulated such that smaller objects are heavier than larger objects” [6], the nervous system appears to encode the *precise shape* of the nonlinear function relating an object’s size to its typical weight, i.e., that objects become denser more quickly the smaller they become following a power function of volume, and these expectations are available to the perceptual system as well as the motor system. This suggests an impressive degree of statistical learning capacity, in that the nervous system has had to extract the non-linear relationship between size and weight from a large of set environmental objects that vary in nearly every conceivable dimension—including shape, color, material (homogenous and heterogeneous), size, weight, and density—and extract the statistical relationship between size and weight buried in the enormously noisy and variable set of data to a remarkable degree of quantitative precision. To our knowledge, this is the first demonstration of quantitative encoding and usage of any joint environmental statistic. The current findings thus inform the field of visuohaptic and visuomotor integration: The predictive step in forward models of motor control is crucial to their ability to demonstrate adaptive and precise motor behavior [1–3].

These findings also have interesting implications for studies of heaviness perception and in particular the size-weight illusion (SWI), in which the smaller of two equally-weighted and similar-looking objects feels heavier than the larger [43] despite no asymmetry in motor force production [33,34]. Evidence suggests that visual and haptic information is combined with prior expectations when lifting novel objects to produce the sensation of heaviness [7–9,44]. To date, studies of the SWI assume, either implicitly or explicitly, that observers expect that differently-sized objects appearing to be made out of the same material will possess the same density [5,7,33]. Our findings demonstrate that this assumption is flawed, since density is not independent of volume for liftable, man-made objects and the nervous system is sensitive to this statistical regularity. It should be noted, however, that even if observers believe smaller objects are denser, they still expect larger ones to be heavier, albeit not by enough to match the size discrepancy; thus, the source of the SWI remains elusive, but it is evident that more investigation is required (a recent review sums up current theories of the SWI and other weight illusions [37]).

Of course, the contribution of density variation itself to heaviness perception has been studied extensively. Researchers have consistently noted that denser objects are perceived as heavier, and that perceived heaviness is a function of an object’s size, shape, and density [25,45–48]. Given the importance of physical density in heaviness perception, it is therefore surprising that prediction of weight based on *predicted* density given an object’s *size* (rather than material) has been largely neglected in studies heaviness perception.

Our results show for the first time that (a) for man-made, liftable objects, density and volume are not independent in the everyday environment; and (b) the human nervous system can represent this complex relationship and abstract from it to generate accurate quantitative expectations about novel objects’ weight relationships. Similarly incorporating quantitative prior knowledge may improve estimates of object weight in artificial systems as well, providing an environmentally-based foundation for the predictive step in forward internal models of motor control [1–3]. Finally, knowledge of these statistics is available to the perceptual system, yet was likely acquired through experience lifting and manipulating objects. Thus, not only can perception influence action [49], but past actions may influence perception as well.

## Supporting Information

S1 Fig Non-log-transformed data. (a) 3-D scanned liftable artificial objects, (b) 3-D scanned natural objects, (c) liftable man-made objects collected by online survey, and (d) artificial but *unliftable* objects collected via online survey.
(TIF)

S1 Dataset Tape measure database (Dataset 1 in S1 Dataset). (XLSX)

S2 Dataset Online object database (Datasets 2 and 5 in S2 Dataset). (XLSX)

S3 Dataset 3-D scanned object database (Datasets 3 and 4 in S3 Dataset). (XLSX)

S4 Dataset Perceptual experiment data. (SAV)
