# Supplementary figures and images for: Smaller = Denser, and the Brain Knows It: Natural Statistics of Object Density Shape Weight Expectations
Source: PLoS One. 2015 Mar 13;10(3):e0119794. doi: 10.1371/journal.pone.0119794 (PMC4358826; doi:10.1371/journal.pone.0119794)

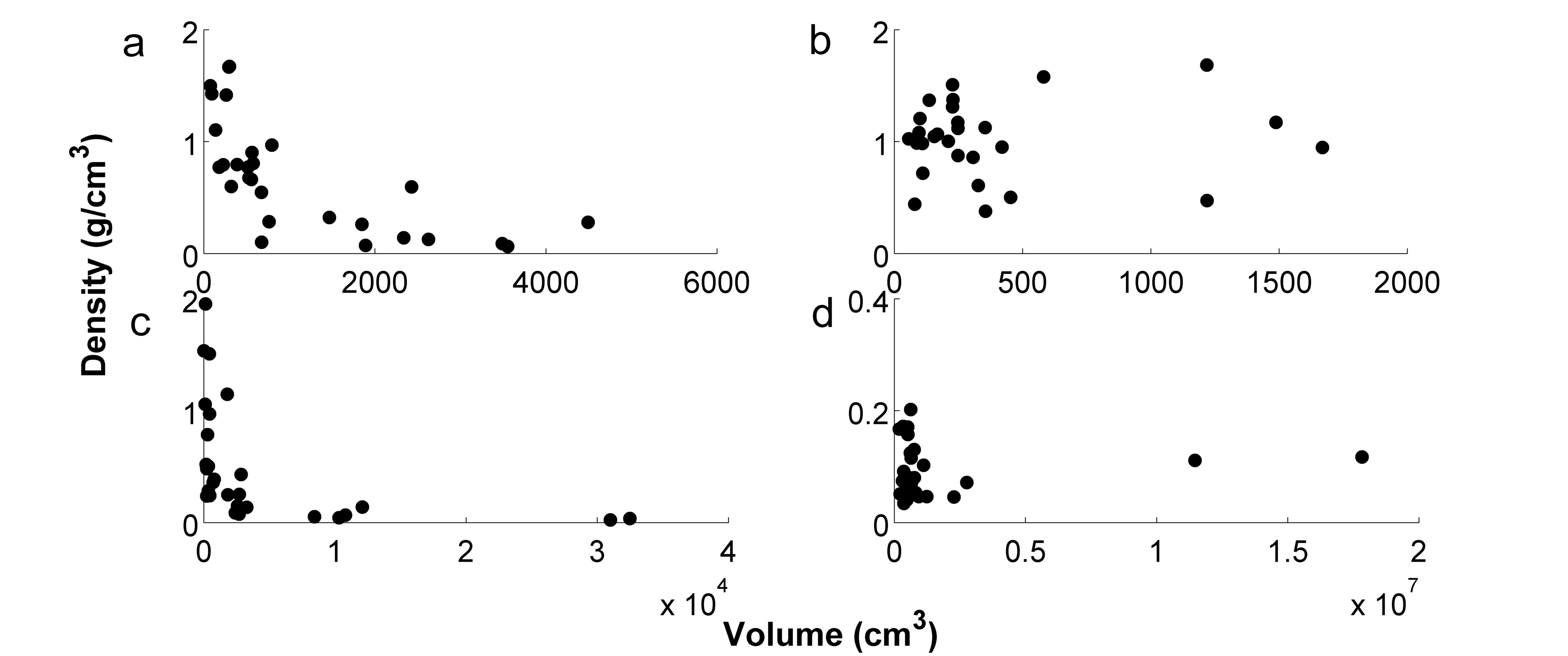

Supplement: S1 Fig — (a) 3-D scanned liftable artificial objects, (b) 3-D scanned natural objects, (c) liftable man-made objects collected by online survey, and (d) artificial but unliftable objects collected via online survey. (TIF) [file pone.0119794.s001.tif]
